# Recovering Actives in Multi-Antitarget and Target Design of Analogs of the Myosin II Inhibitor Blebbistatin

**DOI:** 10.3389/fchem.2018.00179

**Published:** 2018-05-24

**Authors:** Bart I. Roman, Rita C. Guedes, Christian V. Stevens, Alfonso T. García-Sosa

**Affiliations:** ^1^Research Group SynBioC, Department of Green Chemistry and Technology, Faculty of Bioscience Engineering, Ghent University Ghent, Belgium; ^2^Cancer Research Institute Ghent Ghent, Belgium; ^3^Department of Medicinal Chemistry, Faculty of Pharmacy, Research Institute for Medicines (iMed.ULisboa), Universidade de Lisboa Lisbon, Portugal; ^4^Department of Molecular Technology, Institute of Chemistry, University of Tartu Tartu, Estonia

**Keywords:** blebbistatin, fingerprint, ECFP, multitarget, antitarget, myosin II, ATPase, motility

## Abstract

In multitarget drug design, it is critical to identify active and inactive compounds against a variety of targets and antitargets. Multitarget strategies thus test the limits of available technology, be that in screening large databases of compounds vs. a large number of targets, or in using *in silico* methods for understanding and reliably predicting these pharmacological outcomes. In this paper, we have evaluated the potential of several *in silico* approaches to predict the target, antitarget and physicochemical profile of (*S*)-blebbistatin, the best-known myosin II ATPase inhibitor, and a series of analogs thereof. Standard and augmented structure-based design techniques could not recover the observed activity profiles. A ligand-based method using molecular fingerprints was, however, able to select actives for myosin II inhibition. Using further ligand- and structure-based methods, we also evaluated toxicity through androgen receptor binding, affinity for an array of antitargets and the ADME profile (including assay-interfering compounds) of the series. In conclusion, in the search for (*S*)-blebbistatin analogs, the dissimilarity distance of molecular fingerprints to known actives and the computed antitarget and physicochemical profile of the molecules can be used for compound design for molecules with potential as tools for modulating myosin II and motility-related diseases.

## Introduction

Multitarget drug design attempts to rationalize interactions with targets and antitargets. A fine balance is required given that a compound needs to have the right amount of promiscuity, i.e., selectivity. If only one target is hit, an alternative pathway may evolve around the target and the compound may end up lacking efficacy. Too much promiscuity or non-specific interactions, however, will lead to side-effects or toxicity related to antitargets.

Computational methods are central to the ability to predict interactions between compounds and targets given their ability to use a large amount of data on both. They help to prioritize compounds for development or help in target profiling. Several methods can be used, among them structure-based design, as well as filters and bioinformatics approaches (Schneider, 2018).

**Figure 1:**
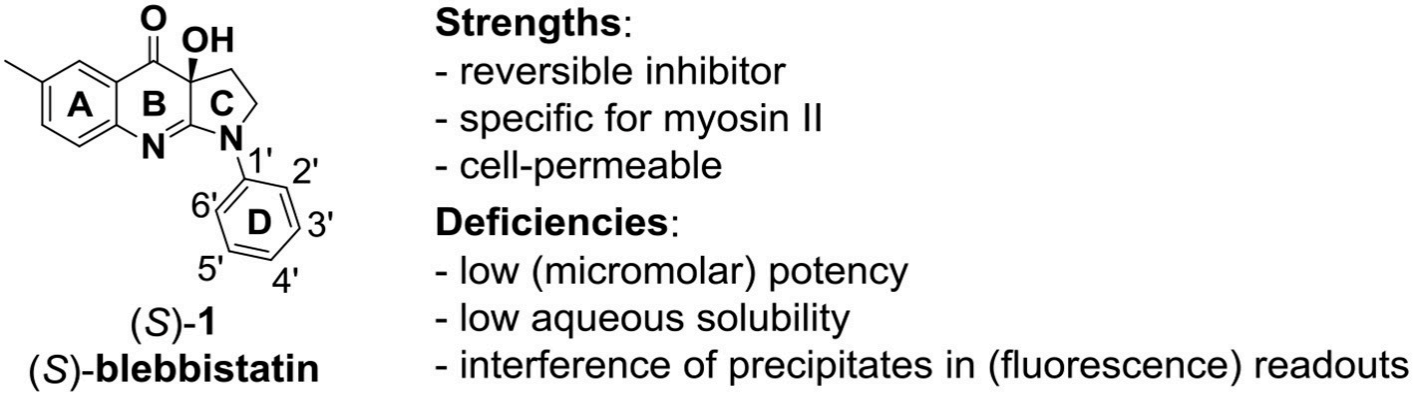
The myosin II ATPase inhibitor (*S*)-**blebbistatin** ((*S*)-**1**): structure, numbering system, strengths and deficiencies.

This paper focuses on the use of such techniques for the multitarget (target and antitargets) design of inhibitors of myosins, the ATP-driven molecular motor proteins of the eukaryotic cell (Sweeney and Houdusse, 2010). The pyrroloquinolinone (*S*)-blebbistatin, (*S*)-**1** in Figure 1, is a cell-permeable, micromolar ATPase cycle inhibitor of myosin II. It is the only available myosin II-specific inhibitor: it does not modulate myosins I, V, and X (Limouze et al., 2004). Since its discovery (Cheung et al., 2001), (*S*)-blebbistatin has therefore been used extensively as a tool for understanding the function of myosin II (Coluccio, 2008; Vicente-Manzanares et al., 2009; Bond et al., 2012), and for dissecting its role in pathological processes such as invasion and in malignant disease (Duxbury et al., 2004; Betapudi et al., 2006; Derycke et al., 2011; Ivkovic et al., 2012), viral infections (Lehmann et al., 2005; Kumakura et al., 2015; Gao et al., 2016), bacterial infections (Lum and Morona, 2014), glaucoma (Zhang and Rao, 2005), progressive renal disease (Si et al., 2010), and methamphetamine use relapse (Young et al., 2016).

(*S*)-blebbistatin ((*S*)-**1)** bears deficiencies that encumber its use in sophisticated biological model systems or as a lead for the development of pharmaceutical tools: its potency is too low (micromolar range) (Verhasselt et al., 2017a,b,c), it is toxic to certain cell lines and organisms (Kolega, 2004; Sakamoto et al., 2005; Mikulich et al., 2012), and it has poor water solubility (Képiró et al., 2012, 2014; Swift et al., 2012; Verhasselt et al., 2017a,b,c). Derivatives with improved aqueous solubility (Várkuti et al., 2016; Verhasselt et al., 2017a,b) and reduced toxicity have been prepared. However, despite the clinical interest in myosin II inhibitors and significant efforts by several groups (Lucas-Lopez et al., 2008; Lawson et al., 2011; Verhasselt et al., 2017a,c), blebbistatin analogs with higher potency have thus far not been prepared. The main reason for this failure is the empirical observation that selectivity and affinity of myosin inhibitors cannot be rationalized from analysis of the residues lining the binding pocket (Sirigu et al., 2016; Verhasselt et al., 2017b,c). Other factors, such as the kinetics of the chemo-mechanical cycle must play an important role in myosin ligand discrimination.

As conventional medicinal chemistry approaches have failed to identify (*S*)-blebbistatin analogs with improved development profiles, we evaluated the potential of a variety of structure-based and ligand-based techniques. Focus was first put on the recovery of active blebbistatin derivatives among a series of analogs, where some methods clearly achieved better outcomes than others. Filters for absorption, distribution, metabolism, and excretion (ADME) profiles and PAINS compounds (non-specific, assay-interfering compounds) were also evaluated. Antitarget effects were studied using a battery of antitarget proteins with physiological significance for myosin inhibitors, including the androgen receptor involved in hormonal systems and skeletal muscle differentiation (Rayment et al., 1993; Wannenes et al., 2008), the pregnane X receptor (PXR) involved in efflux of xenobiotics, sulfotransferase (SULT) and cytochrome P450 (CYP) isoforms involved in human metabolism of substances.

## Methods

### Compounds

The library under study contains 19 compounds (Table 1). Structure-activity data were taken from our earlier reports on blebbistatin analogs (Verhasselt et al., 2017a,b,c). These activity data were obtained using one assay protocol and were collected by one observer. Synthetic protocols and generation of ATPase inhibitory activity data against rabbit skeletal-muscle myosin in an in-house developed assay are described in these papers. The studied library contains both compounds that are active and inactive against myosin II ATPase activity.

**Table 1:** Studied compounds: (*S*)-blebbistatin ((*S*)-**1**) and analogs, inhibitory potency against rabbit skeletal muscle myosin II ATPase activity and references.

**Compound**		**IC_50_ (μM)**	**References**
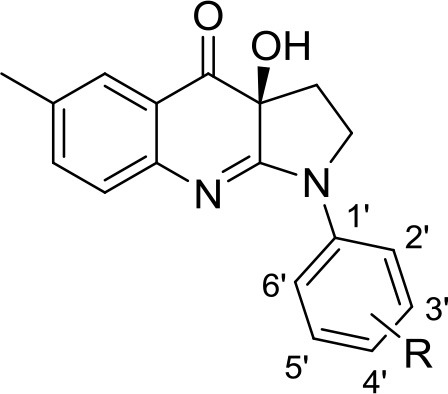	R =		
(*S*)-**1** ((*S*)-**Blebbistatin**)	H	2.16	Verhasselt et al., 2017b
(*S*)-**2**	3′-CN	48.5	Verhasselt et al., 2017b
(*S*)-**3**	3′-COOH	≥300	Verhasselt et al., 2017b
(*S*)-**4**	3′-CONH_2_	≥300	Verhasselt et al., 2017b
(*S*)-**5**	3′-OAllyl	9.41	Verhasselt et al., 2017a
(*S*)-**6**	3′-OH	19.3	Verhasselt et al., 2017b
(*S*)-**7**	3′-OAcryl	57.6	Verhasselt et al., 2017a
(*S*)-**8**	3′-NH_2_	14.1	Verhasselt et al., 2017b
(*S*)-**9**	3′-OPropanoyl	23.5	Verhasselt et al., 2017a
(*S*)-**10**	3′-NHPropanoyl	≥300	Verhasselt et al., 2017a
(*S*)-**11**	3′-NHAcryl	≥300	Verhasselt et al., 2017a
(*S*)-**12**	4′-OAllyl	0.94	Verhasselt et al., 2017a
(*S*)-**13**	4′-OH	13.5	Verhasselt et al., 2017a
(*S*)-**14**	4′-OBn	≥300	Verhasselt et al., 2017a
(*S*)-**18**	4′-NH_2_	9.22	Várkuti et al., 2016
(*S*)-**19**	4′-NO_2_	1.96	Képiró et al., 2014
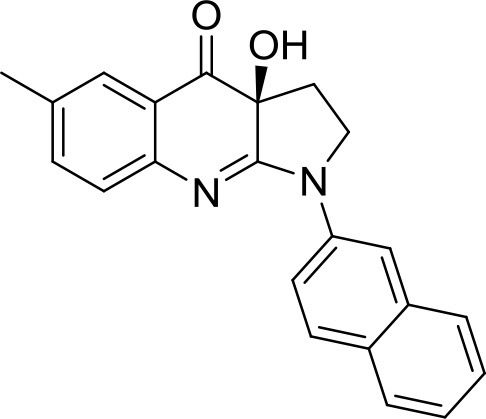			
(*S*)-**15**		≥300	Verhasselt et al., 2017b
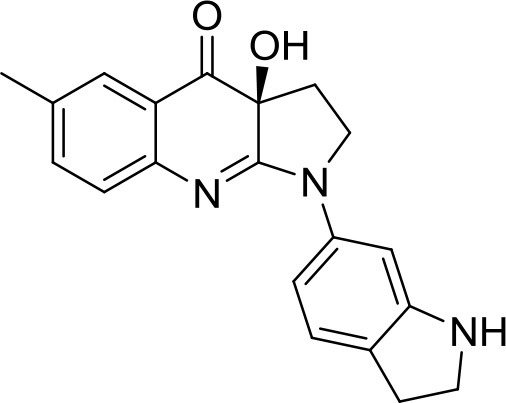			
(*S*)-**16**		19.0	Verhasselt et al., 2017b
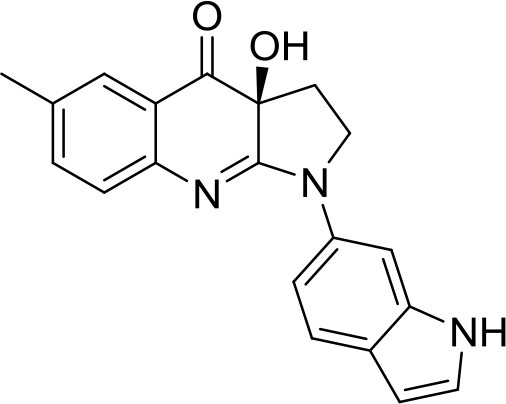
(*S*)-**17**		17.8	Verhasselt et al., 2017b

### Docking

The crystal structure of *Dictyostelium discoideum* myosin II, 1YV3 (Allingham et al., 2005), was selected and downloaded from the Protein Data bank (Berman et al., 2000). The resolution of the crystal structure determination was 2.00 Ångström and contained the co-crystallized ligand (*S*)-blebbistatin ((*S*)-**1**, BIT), also called (-)-1-phenyl-1,2,3,4-tetrahydro-4-hydroxypyrrolo[2,3-b]-7-methylquinolin-4-one, or (*S*)-(3a)-hydroxy-6-methyl-1-phenyl-3,3a-dihydro-1H-pyrrolo[2,3-b]quinolin-4(2H)-one. The sequences of rabbit (*Oryctolagus cuniculus*) and *D. discoideum* were aligned and a strong agreement of over 81.8% identity in the residues of the binding site was found. The binding site (site AC5

in pdb) consists of Phe 239, Gly 240, Tyr 261, Leu 262, Leu 263, Ser 456, Glu 467, Cys 470, Tyr 634, Gln 637, and Leu 641 for *D. discoideum*, which correspond to Phe 239, Gly 240, Tyr 261, Leu 262, Leu 263, Asp 456, Glu 467, Cys 470, Phe 634, Gln 637, and Leu 641 in rabbit myosin II heavy chain. The structure alignment is available in Table S1. Crucial residues are conserved in the binding site of myosin II heavy chain between these species, as well as with human, with the rest of the pocket consisting of hydrophobic areas that are also similar for these species. Also important, the same experimental response to blebbistatin was observed by the enzyme of all three species (Table S2). Docking calculations with and without crystallographic water molecules were conducted using GOLD (v5.22) (Jones et al., 1997) and Glide (Glide, 2017). For Glide, the protein structure was prepared with Maestro, and the XP scoring function, flexible ligand, and rigid protein settings were used, as well as no Epik penalties (state penalties based on predicted populations in solution for the structures it generates) were applied. VdW radii of protein atoms was scaled by 1 (i.e., not scaled), and the charge cutoff for polarity was 0.25, with the grid containing 10 Å in x, y, and z, centered on 22.37, 38.35, 37.18, for the inner box, and 30 Å in x, y, and z for the outer box. For GOLD, the structure of myosin II was prepared with MOE2016 software. The docking protocol (protein structure and docking conditions) was validated by re-docking the crystallographic ligand. Blebbistatin analogs were built and their energy was minimized using MOE2016 software and then docked into the allosteric binding site in the myosin II S1 motor domain, using the validated docking protocols. For the docking simulations with GOLD a search space sphere with a radius of 15 Å was defined as the binding pocket of myosin II, centered on the crystallographic coordinates of the oxygen atom of Ser456 residue. Docking calculations were performed using the following parameters: number of islands = 5, population size = 100, number of operations = 100,000, a niche size = 2, and a selection pressure = 1.1. All H-bond donors/acceptors were treated as solvent accessible. For each compound 1,000 runs were performed. Flip of pyramidal N, amide bonds, and ring corners were allowed. To score the compounds, the scoring function was used after being validated. The search efficiency was set to 100%. Ten top docking poses were retained for each compound.

### Antitargets

The battery of antitargets that have physiological significance for experiments involving blebbistatin was assessed with three different docking programs (Schrödinger Glide XP, Glide, 2017, Autodock4, Morris et al., 1998, and Autodock Vina, Trott and Olson, 2010), each with its own scoring function, as described in previous work (García-Sosa and Maran, 2014). The collected interactions against PXR (Watkins et al., 2003), SULT (Lu et al., 2005), CYP 2A6 (Yano et al., 2005), CYP 2C9 (Williams et al., 2003), and CYP 3A4 (Yano et al., 2004) were calculated and then scored and visualized. The structure of the androgen receptor, 1T7R (Hur et al., 2004), with a resolution of 1.4 Ångströms, was downloaded from the Protein Databank (Berman et al., 2000). It contained the co-crystallized ligand dihydrotestosterone (DHT), a known active. Hydrogens were added and the proteins' structure titrated with Maestro^1^

### ADME

FAF (*Free ADME-Tox Filtering Tool)* filters were used to calculate ADME parameters for the compounds (Lagorce et al., 2008). Briefly, the filters are based on searches of substructures within ligands and previous knowledge of PAINs, reactive groups, solubility, Lipinski's, Veber's, and Egan's rules, among others.

### Fingerprints

Dissimilarity distance calculations between compounds were carried out using extended connectivity fingerprints (ECFP), as implemented in ChemAxon^2^. Compounds were separated into a group of actives (experimental IC_50_ < 10 μM) and a group of inactives (IC_50_ > 10 μM). Dissimilarity distances were calculated by Tanimoto coefficients between individual compounds, and also between each compound and the average distance to all the actives (both including and excluding (called “actives-self”) said compound if in that group) or to all the inactives.

## Results and discussion

The structures of the compounds under study, i.e., (*S*)-blebbistatin ((*S*)-**1**) and a series of structural analogs, are presented in Table 1 (Verhasselt et al., 2017a,b).

### Docking

**Figure 2:**
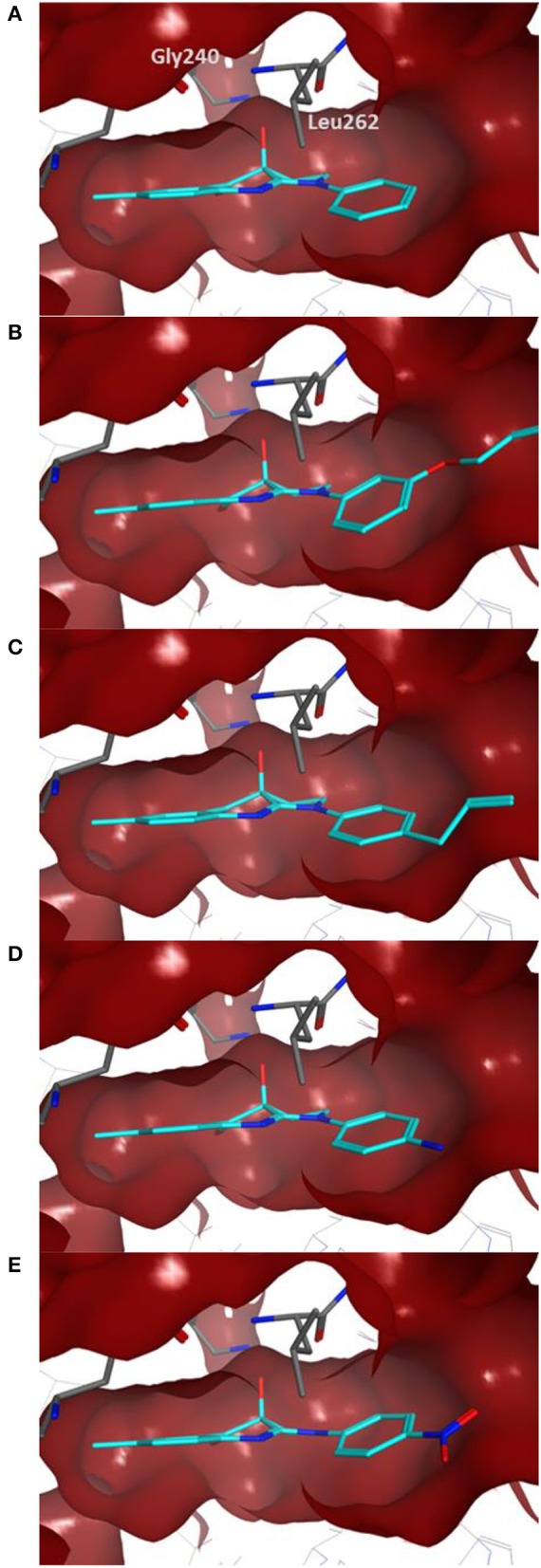
Best docking poses for five (*S)-*blebbistatin analogs (cyan) inside the myosin II binding pocket (in red surface and gray sticks) without water molecules obtained using GOLD 5.22 software: **(A)** (*S*)-**1**; **(B)** (*S*)-**5**; **(C)** (*S*)-**12**; **(D)** (*S*)-**18**; **(E)** (*S*)-**19**.

Docking of the blebbistatin analogs under study (Table 2, Figure 2) was carried out with and without crystallographic waters in the Myosin II binding site using GOLD 5.22 and Glide XP software. The docked blebbistatin library occupied the same region and displayed the same binding mode as the co-crystallized ligand. Figure 2 shows the best binding poses obtained for five (*S*)-blebbistatin analogs (results obtained without crystallographic waters). All docked (*S*)-blebbistatin analogs occupying the same region of the binding pocket, had orientations highly resembling the crystallographic ligand, and were able to establish two hydrogen bonds between their OH moiety and Leu 262 and Gly 240 in the myosin II binding pocket. From these docking results, it is not possible to anticipate the structural features responsible for the loss or the improvement of the inhibitory activity (Table 2) of (S)-blebbistatin analogs. Also, the docking scores are not able to differentiate between actives and inactives, e.g., in the absence of waters for the most active compound (*S*)-**12** (IC_50_ = 0.94 μM), we obtain a score of 104.7 with GOLD (or −12.49 with Glide), but for the second most active compound (*S*)-**19**, (IC_50_ = 1.96 μM), the lowermost score of 87.4 is obtained with GOLD (or −12.19 with Glide). No correlation was found between scores and activity.

**Table 2:** Experimental inibitory activities and docking Scores (with and without crystallographic waters) obtained with Glide XP and GOLD Chemplp.

**Compound^a^**	**IC_50_ (μM)**	**Glide XP score with waters^b^**	**Glide XP score without waters^b^**	**ChemPLP without waters^c^**	**ChemPLP with waters^c^**
(*S*)-**1**	2.16	−13.07	−12.65	95.5	108.23
(*S*)-**2**	48.5	−12.24	−12.79	95.9	108.67
(*S*)-**3**	≥300	−13.91	0	93.6	106.37
(*S*)-**4**	≥300	−13.55	−13.03	92.9	105.62
(*S*)-**5**	9.41	−13.80	−12.15	106.88	119.55
(*S*)-**6**	19.3	−13.91	−13.03	97.9	110.65
(*S*)-**7**	57.6	−13.42	0	107.18	119.82
(*S*)-**8**	14.1	−11.93	−14.04	97.9	110.73
(*S*)-**9**	23.5	−12.97	−12.45	106.68	119.46
(*S*)-**10**	≥300	−13.86	0	108.31	120.97
(*S*)-**11**	≥300	−13.72	0	110.36	123.04
(*S*)-**12**	0.94	−13.01	−12.49	104.7	117.47
(*S*)-**13**	13.53	−12.01	−13.14	95.22	107.9
(*S*)-**14**	≥300	−13.30	0	116.16	128.92
(*S*)-**15**	≥300	−12.70	0	102.39	115.08
(*S*)-**16**	19.04	−12.16	−12.83	103.49	116.19
(*S*)-**17**	17.81	−12.45	−12.83	100	112.68
(*S*)-**18**	9.22	−12.19	−13.23	95.01	107.73
(*S*)-**19**	1.96	−12.05	−12.19	87.43	100.08

a Structures of compounds (S)-**1** to (S)-**19** are shown in Table 1.

b Glide XP score (kcal/mol).

c *ChemPLP (GOLD fitness score)*.

**Figure 3:**
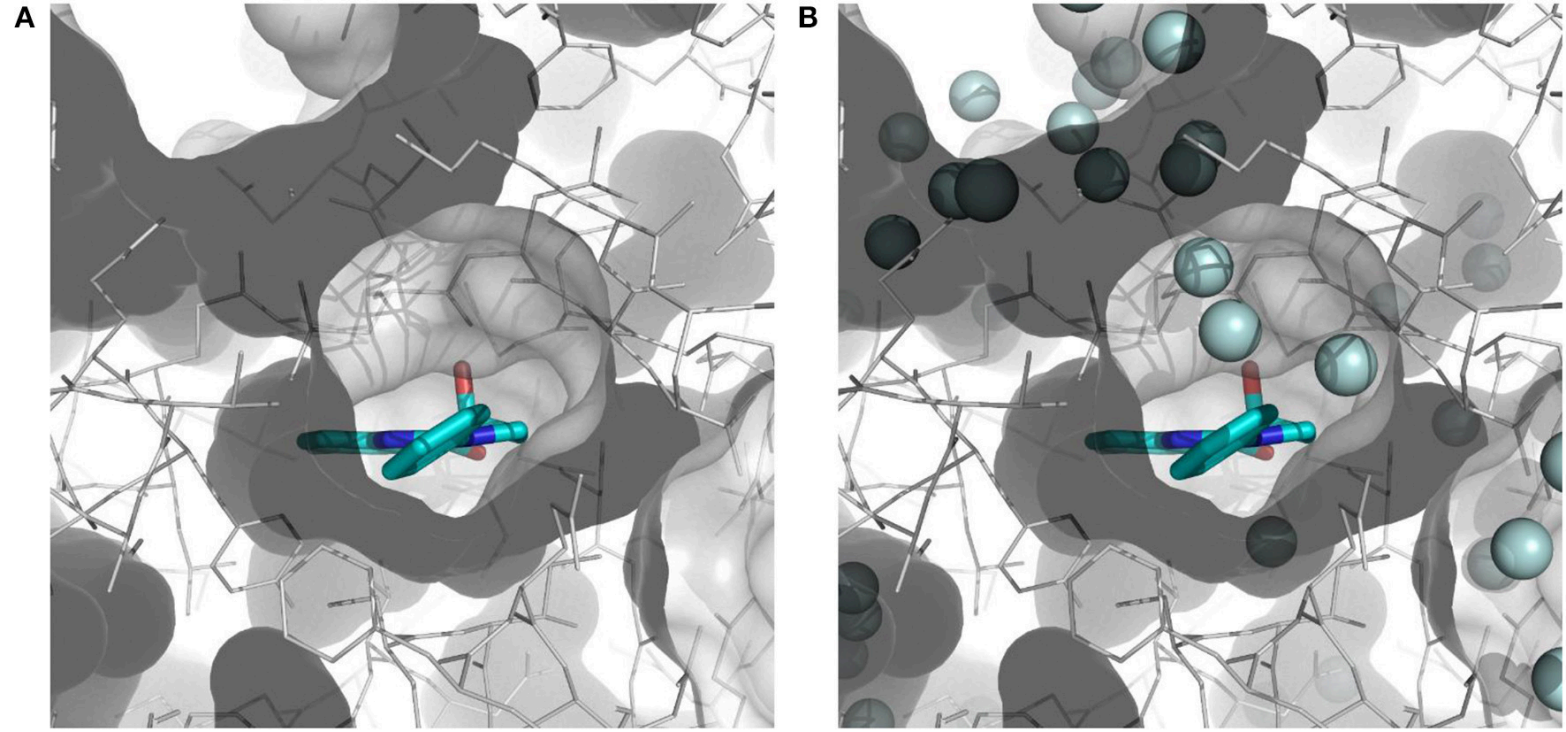
Binding site of myosin II (in white) with co-crystallized ligand (*S*)-blebbistatin (BIT, cyan): **(A)** without water molecules, and **(B)** with explicit, crystallographically-observed water molecules (in light blue).

The importance of including crystallographic waters in the analysis is illustrated in Figure 3. From these illustrations, it is also clear that the binding site is very tight. The path toward it from the surface of the protein is moreover very constrained, with a nearly occluded mouth leading to a thin channel into the small binding pocket.

**Figure 4:**
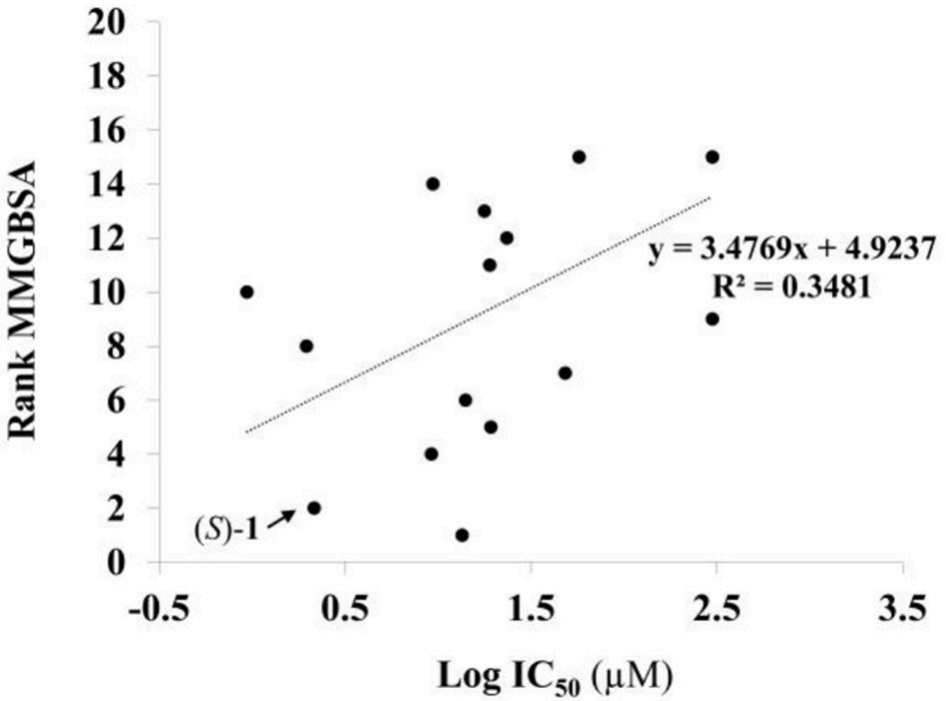
Docking with crystallographic waters of study compounds: correlation of log IC_50_ values (measured) and rank MMGBSA (calculated).

**Figure 5:**
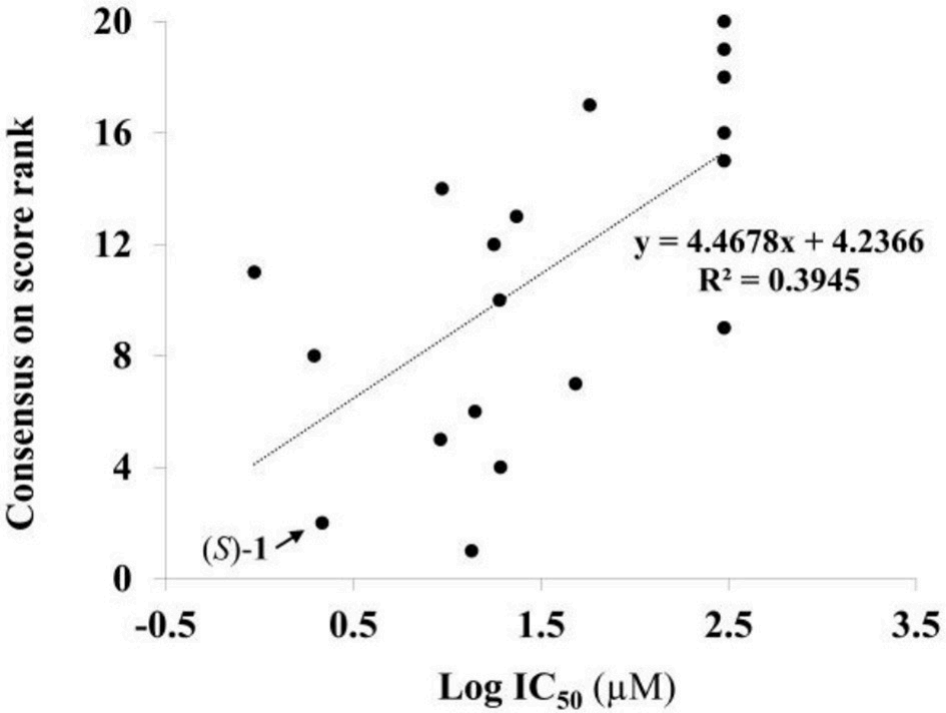
Docking with crystallographic waters of study compounds: correlation of log IC_50_ values (measured) and consensus on score rank (calculated).

Given the above negative individual results and binding site considerations, consensus methods were evaluated to find correlations between experimental activity data and docking scores. Docking results of the library of (*S*)-blebbistatin analogs with crystallographic waters using a series of different programs and scoring functions were explored. The outcomes are summarized in Table 3. Included are all compounds from Table 1 with their *in silico* optimized geometries. Parent compound (*S*)-blebbistatin is included both with its geometry as a co-crystallized ligand (entry BIT) and with its *in silico* optimized geometry (entry (*S*)-**1**). When comparing these results to experimental log IC_50_ values, no strong correlations were found for ranks calculated by MMGBSA (Figure 4), and for ranks by Consensus on score (Figure 5).

**Table 3:** Docking results with water molecules.

**Compound^a^**	**Glide XP**		**Glide SP**		**MMGBSA_dG_Bind**		**Autodock 4**		**Autodock Vina**		**Consensus**			
	**Score^b^**	**Rank**	**Score^c^**	**Rank**	**Score^d^**	**Rank**	**Score^e^**	**Rank**	**Score^f^**	**Rank**	**On score**	**On score rank**	**On rank**	**Rank on rank**
BIT	−12.65	9	−12.14	8	−73.50	3	−11.35	2	−12.9	2	−24.51	3	4.8	2
(*S*)-**18**	−13.23	2	−12.62	5	−70.28	4	−10.96	7	−12.2	8	−23.86	5	5.2	3
(*S*)-**19**	−12.19	13	−11.94	11	−63.91	8	−9.8	14	−12.3	6	−22.03	8	10.4	11
(*S*)-**1**	−12.62	10	−12.12	9	−75.25	2	−11.11	6	−12.3	6	−24.68	2	6.6	7
(*S*)-**2**	−12.79	8	−12.63	4	−67.36	7	−10.61	9	−11.4	11	−22.96	7	7.8	8
(*S*)-**3**	0	15	0	15	0	15	−9.32	17	−12.4	4	−4.34	16	13.2	14
(*S*)-**4**	−13.03	4	−12.34	7	−58.51	9	−10.18	11	−11.5	10	−21.11	9	8.2	9
(*S*)-**5**	−12.15	14	−9.62	14	−40.89	14	−10.11	12	−9.9	14	−16.53	14	13.6	16
(*S*)-**6**	−13.03	5	−12.69	3	−69.83	5	−11.2	4	−12.8	3	−23.91	4	4	1
(*S*)-**7**	0	15	0	15	0	15	−9.73	15	−9.5	15	−3.85	17	15	17
(*S*)-**8**	−14.04	1	−12.73	2	−68.77	6	−11.15	5	−11.3	12	−23.60	6	5.2	3
(*S*)-**9**	−12.45	12	−11.82	12	−48.96	12	−9.68	16	−9.5	15	−18.48	13	13.4	15
(*S*)-**10**	0	15	0	15	0	15	−8.72	18	−8.2	18	−3.38	18	16.2	18
(*S*)-**11**	0	15	0	15	0	15	−8.16	19	−7.8	19	−3.19	19	16.6	19
(*S*)-**12**	−12.49	11	−11.67	13	−54.21	10	−9.84	13	−9.4	17	−19.52	11	12.8	13
(*S*)-**13**	−13.14	3	−13.01	1	−77.04	1	−10.64	8	−10.4	13	−24.85	1	5.2	3
(*S*)-**14**	0	15	0	15	0	15	−6.34	20	−5.9	20	−2.45	20	17	20
(*S*)-**15**	0	15	0	15	0	15	−11.34	3	−12.4	4	−4.75	15	10.4	11
(*S*)-**16**	−12.83	6	−12.09	10	−51.78	11	−10.51	10	−11.9	9	−19.82	10	9.2	10
(*S*)-**17**	−12.83	7	−12.52	6	−45.63	13	−11.78	1	−13	1	−19.15	12	5.6	6

a Structures of compounds (S)-**1** to (S)-**19** are shown in Table 1.

b Glide XP score (XP, kcal/mol).

c Glide SP score (SP, kcal/mol).

d Molecular Mechanics Generalized Born/Surface Area Binding energy (MMGBSA_dG_Bind, kcal/mol).

e Autodock4 binding energy (AD4, kcal/mol).

f *Autodock Vina score (Vina, kcal/mol)*.

Based on these results, structure-based methods for myosin II seem to be limited in their ability to predict active compounds. Only some of the largest molecules that do not fit into the binding pocket are correctly rejected by some of the docking programs. The total volume of the binding site, excluding crystallographically-observed water molecules, is 417.77 Å^3^ according to the SiteMap module of the Schrödinger package. This is indeed close to the volume of the largest ligand, (*S*)-**14**, at 318.0 Å^3^.

**Figure 6:**
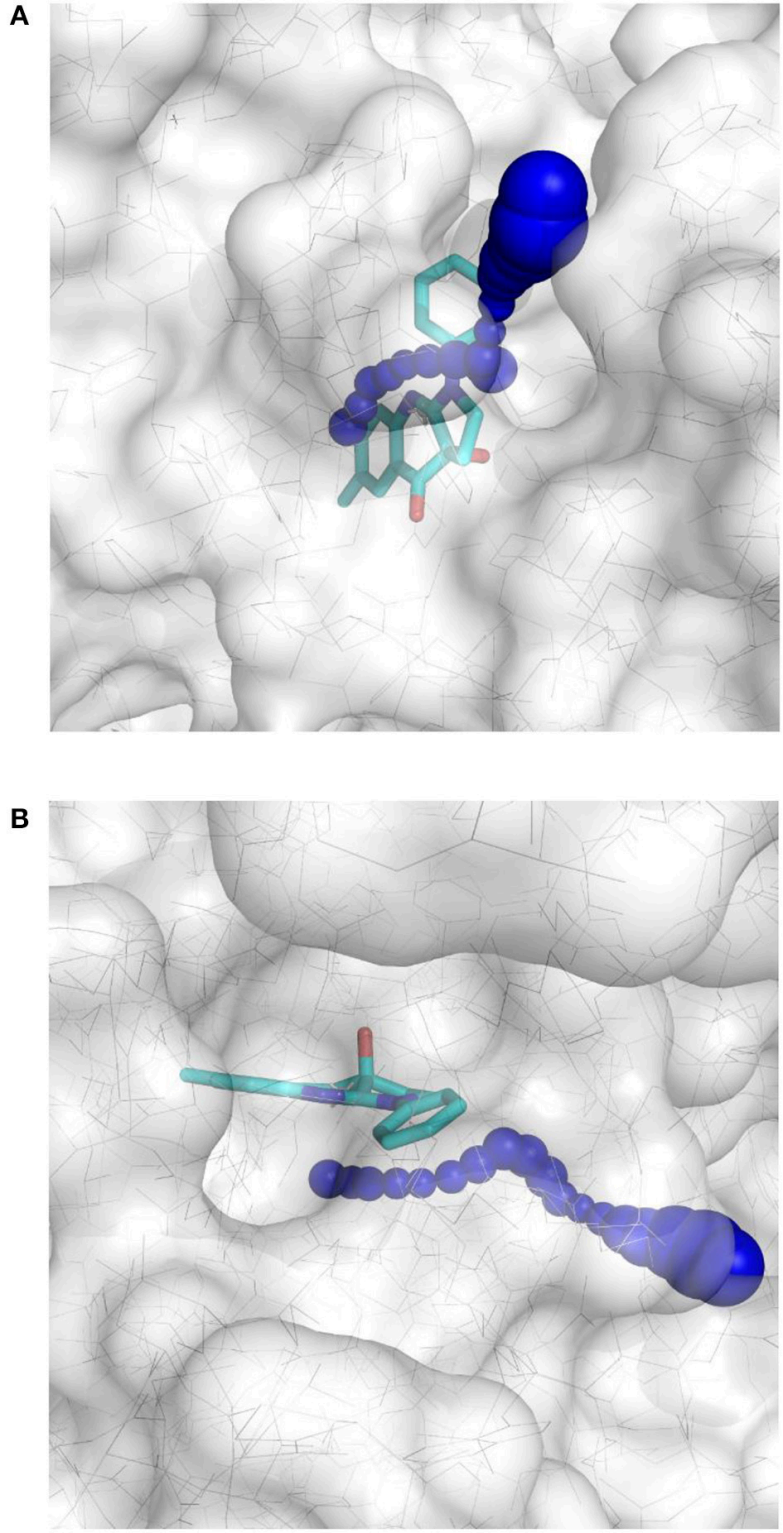
Top-view **(A)** and side-view **(B)** of the tight tunnel (blue spheres) from the protein surface (in white) leading to the complexed ligand blebbistatin (in cyan).

This result obtained for the majority of the compounds regardless of the docking program may reflect the aforementioned difficult access to the binding site. The protein structure complex with (*S*)-blebbistatin was therefore studied using CAVER (Pavelka et al., 2016), an algorithm for the detection of tunnels in macromolecules (Figure 6). Difficulty of access to the binding site was confirmed, revealing a tight tunnel that leads to the ligand as shown in Figures 6A,B. This tunnel is lined by residues Arg 238, Glu 264, Ser 266, Arg 267, Phe 270, Ser 272, Glu 275, Ser 456, Glu 459, Phe 461, Val 463, Ser 465, Glu 467, Gln 468, Cys 470, Ile 471, Thr 474, Lys 587, Tyr 634, Leu 262, Leu 263, Gln 271, Asn 464, Phe 466, Leu 469, and Asn 472, starting from the surface of the protein toward the inside. These residues may well be involved in limiting access of ligands to the binding site.

### Fingerprints

Chemical fingerprints describe in a digital manner a molecular structure. ECFPs include a chemical awareness of substructures in a compound due to the atom-type description of the neighborhood of each atom in a molecule. The ECFPs for our compound library were calculated and the distance between them was computed. The results of the distances between fingerprints for the study compounds as well as between each compound and the two groups of compounds (actives: IC_50_ < 10 μM; inactives: IC_50_ >10 μM) are shown in Table 4.

**Table 4:** Distance between chemical fingerprints for each compound to the averages of active ((*S*)-**5**, (*S*)-**12**, (*S*)-**18**, and (*S*)-**19**) and inactive compounds^a^.

**Compound**	**IC_50_ μM)**	**Class**	***(S)*-1**	***(S)*-5**	***(S)*-12**	***(S)*-19**	***(S)*-18**	**Actives**	**Actives-self^b^**
(*S*)-**1**	2.16	Active	0	0.339	0.3214	0.2692	0.2083	0.2276	0.2845
(*S*)-**2**	48.5	Inact.	0.2642	0.3692	0.4308	0.3934	0.3214	0.3558	
(*S*)-**3**	300	Inact.	0.2642	0.3692	0.4062	0.3667	0.3214	0.3455	
(*S*)-**4**	300	Inact.	0.2778	0.3788	0.4394	0.4032	0.3333	0.3665	
(*S*)-**5**	9.41	Active	0.339	0	0.1695	0.4	0.4127	0.2642	0.3303
(*S*)-**6**	19.3	Inact.	0.2041	0.3279	0.3934	0.3509	0.3019	0.3156	
(*S*)-**7**	57.6	Inact.	0.3103	0.25	0.3385	0.4	0.3871	0.3372	
(*S*)-**8**	14.1	Inact.	0.22	0.3387	0.4032	0.3621	0.2157	0.3079	
(*S*)-**9**	23.5	Inact.	0.3158	0.2812	0.3939	0.4062	0.3934	0.3581	
(*S*)-**10**	300	Inact.	0.3276	0.3433	0.403	0.4394	0.4032	0.3833	
(*S*)-**11**	300	Inact.	0.35	0.3623	0.4203	0.4559	0.4219	0.4021	
(*S*)-**12**	0.94	Active	0.3214	0.1695	0	0.3051	0.3158	0.2224	0.2779
(*S*)-**13**	13.53	Inact.	0.1915	0.4032	0.3036	0.25	0.1875	0.2672	
(*S*)-**14**	300	Inact.	0.2407	0.3731	0.2787	0.3443	0.3276	0.3129	
(*S*)-**15**	300	Inact.	0.22	0.3906	0.377	0.3333	0.283	0.3208	
(*S*)-**16**	19.04	Inact.	0.3393	0.4493	0.4154	0.377	0.3333	0.3829	
(*S*)-**17**	17.81	Inact.	0.3148	0.4328	0.3968	0.3559	0.3091	0.3619	
(*S*)-**18**	9.22	Active	0.2083	0.4127	0.3158	0.2642	0	0.2402	0.3155
(*S*)-**19**	1.96	Active	0.2692	0.4	0.3051	0	0.2642	0.2477	0.2944
Inactives			0.2743	0.3606	0.3814	0.3672	0.3165		

a Structures of compounds (S)-**1** to (S)-**19** are shown in Table 1.

b *Actives-self, Calculation with actives excluded themselves from the active group during the calculation, i.e., distance to the average of all the other actives*.

After encoding chemical structures, their dissimilarity distance was calculated, with the distance to itself, i.e., identity = 0. Interesting results were found. All actives are closer to the other actives than to the inactive compounds, i.e., they had smaller dissimilarity distances to the averages. This was also the case when actives are excluded themselves from the active group during the calculation, i.e., distance to all the other actives, called “actives-self” in Table 4, with the exception of (*S*)-blebbistatin (*S*)-**1**. Similarity to the active compounds implies a greater affinity for the specific targets of the known actives. In this study, such compounds could thus exhibit specificity toward inhibition of myosin II. Importantly, among the inactive compounds, those molecules that were borderline active, i.e., had measured IC_50_ values of 14.1 μM ((*S*)-**8**) and 13.53 μM ((*S*)-**13**), were also the closest (least dissimilar) to the active chemicals.

**Figure 7:**
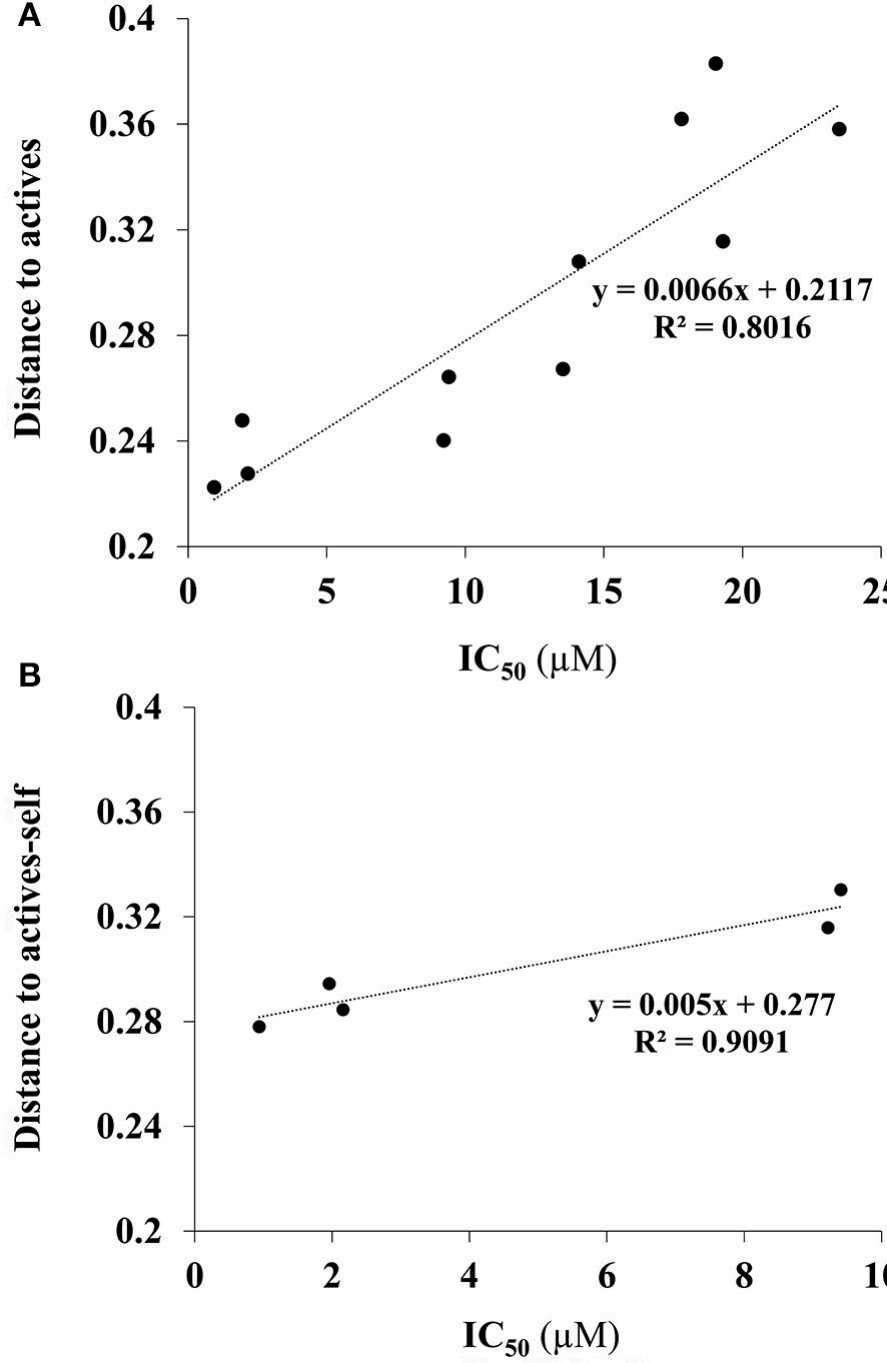
Correlation of IC_50_ values (measured) and average chemical fingerprint distance to actives (calculated): **(A)** For all compounds with an IC_50_ value < 45 μM; **(B)** for all actives (IC_50_ < 10 μM), to actives without that particular compound included.

In the dataset, several inactive compounds possess experimental IC_50_ values ≥ 300 μM. The exact numerical value have not been determined because of solubility limitations and the exact IC_50_ in these instances has no significance. Therefore, correlations with fingerprint dissimilarity distances were investigated in more detail for only those compounds with an IC_50_ value < 45 μM. A good correlation was found between the chemical fingerprint dissimilarity distances to the average of the actives with the experimental IC_50_ values for the set with IC_50_ values < 45 μM (Figure 7A). For the subset of active compounds (IC_50_ < 10 μM), a strong correlation was also obtained when comparing experimental IC_50_ values of individual active compounds with its chemical fingerprint distance to the subset of actives, even without that active compound included (“actives-self,” Figure 7B). In sum, the chemical fingerprint dissimilarity distance calculation may be useful for selecting, solely from structure, active blebbistatin analogs among a set of hypothetical analogs.

### ADME and antitargets

**Figure 8:**
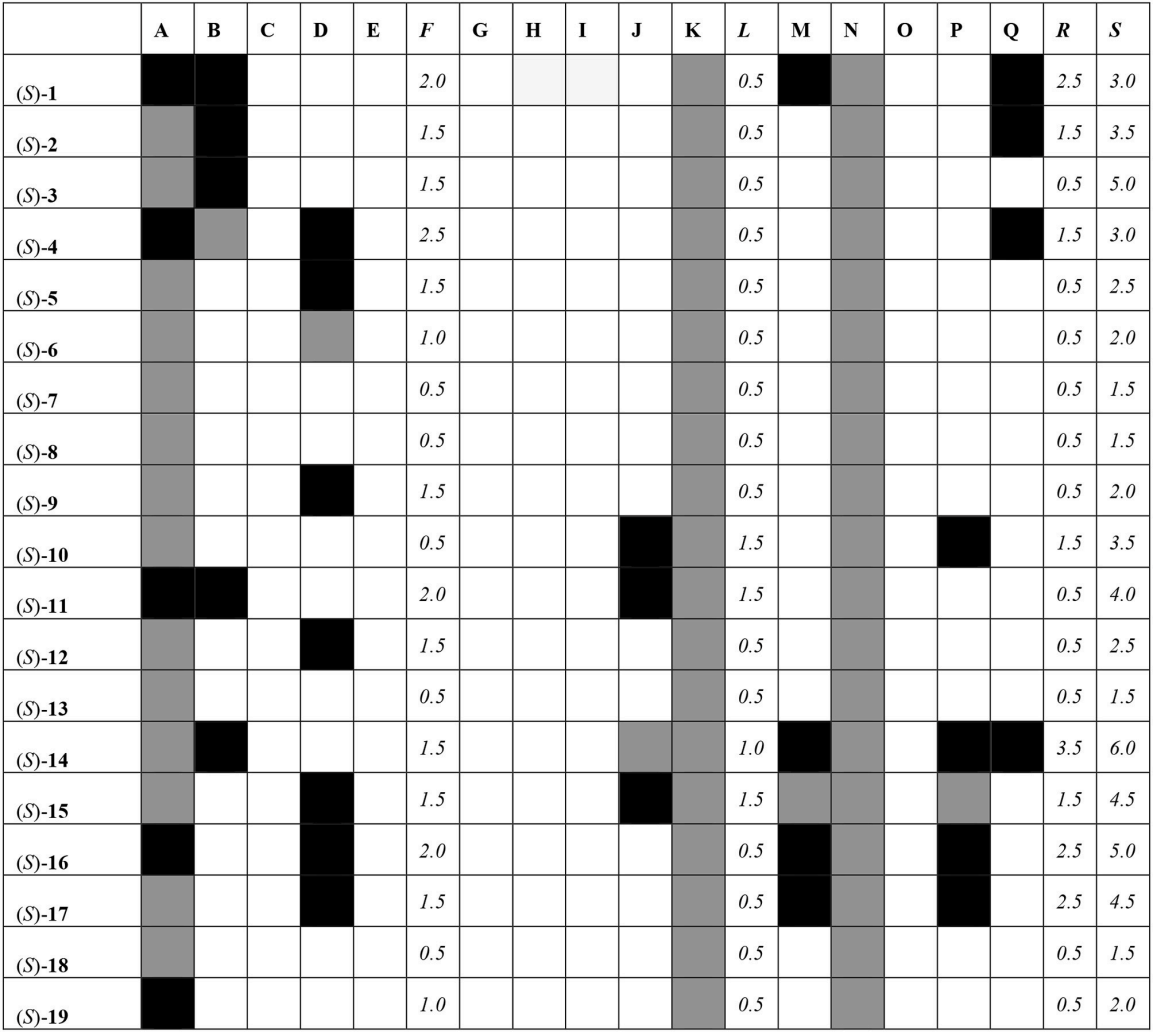
Interaction matrix between proposed ligands and antitargets. Color code: black = 1.0; gray = 0.5, white = 0.0. Columns: *A*, Glide XP PXR; *B*, Glide XP SULT; *C*, Glide XP CYP 2A6; *D*, Glide XP CYP 2C9; *E*, Glide XP CYP 3A4; *F*, Glide XP Total; *G*, Autodock 4 PXR; *H*, Autodock 4 SULT; *I*, Autodock 4 CYP 2A6; *J*, Autodock 4 CYP 2C9; *K*, Autodock 4 CYP 3A4; *L*, Autodock 4 Total; *M*, Vina PXR; *N*, Vina SULT; *O*, Vina CYP 2A6; *P*, Vina CYP 2C9; *Q*, Vina CYP 3A4; *R*, Vina Total; *S*, Grand Total.

All of the compounds under study were docked and interactions were calculated against a battery of antitargets that have physiological significance for experiments involving blebbistatin. Interactions were collected against the pregnane X receptor (PXR), sulfotransferase (SULT), and cytochrome P450 (CYP) isoforms 2A6, 2C9, and 3A4. Three different docking programs were used: Schrödinger Glide XP, Autodock 4, and Autodock Vina. The results are shown in Figure 8, where compounds with interactions stronger than 0.5 kcal/mol than that recorded for the co-crystallized ligand are shaded in black, interactions weaker than 0.5 kcal/mol are shaded in white, and those within 1 kcal/mol (i.e., ±0.5 kcal/mol from that of the co-crystallized ligand) are shaded in gray.

From Figure 8, it can be seen that most compounds interact with PXR, SULT, and CYP 2C9 and 3A4. The compound that hit the most number of anti-targets was (*S*)-**14**, while several others had lower interaction profiles. The most active compounds according to the fingerprint method, i.e., (*S*)-**1**, (*S*)-**5**, (*S*)-**12**, (*S*)-**18**, and (*S*)-**19**, had low interaction profiles against the anti-targets with total scores mostly lower than 3.0.

All the compounds were docked against the androgen receptor structure 1T7R. The results are shown in Table 5. All of the compounds had interaction energies weaker than the active control dihydrotestosterone. The compounds that had interaction energies weaker than −7 kcal/mol were (*S*)-**10**, (*S*)-**11**, (*S*)-**12**, (*S*)-**14**, (*S*)-**15**, (*S*)-**16**, (*S*)-**17**, and (*S*)-**19**. Among the active compounds, (*S*)-**12** and (*S*)-**19** had the lowest interactions against this receptor, which may help avoid this hormonal target and system.

**Table 5:** Docking scores with the Androgen receptor^a^.

**Compound**	**Androgen receptor score (kcal/mol)**
(*S*)-**1**	−8.43
(*S*)-**2**	−8.87
(*S*)-**3**	−7.61
(*S*)-**4**	−8.37
(*S*)-**5**	−8.08
(*S*)-**6**	−8.76
(*S*)-**7**	−8.07
(*S*)-**8**	−8.04
(*S*)-**9**	−7.98
(*S*)-**10**	−5.73
(*S*)-**11**	−5.34
(*S*)-**12**	−6.88
(*S*)-**13**	−8.21
(*S*)-**14**	–
(*S*)-**15**	–
(*S*)-**16**	–
(*S*)-**17**	–
(*S*)-**18**	−7.18
(*S*)-**19**	–
dihydrotestosterone	−11.91

a *Structures of compounds (S)-**1** to (S)-**19** are shown in Table 1*.

ADME results from FAF filters showed that all of the compounds pass Lipinksi's rule of five, as well as Veber and Egan bioavailability rules. All of the compounds passed the PAINS filters, which detect non-specific, assay-interfering compounds, except (*S*)-**18** with a warning on *ortho*-aniline. All compounds passed Lilly's filters, except warnings for (*S*)-**7** (risk as Michael acceptor), (*S*)-**9** (phenolic ester or carbamate), and (*S*)-**11** (risk as Michael acceptor). Leeson values are normally under 1.0 for drugs (Leeson and Springthorpe, 2007). For the active compounds, (*S*)-**5**, (*S*)-**12**, and (*S*)-**19** compounds had interesting calculated Leeson values of −0.39, −0.39, and 0.09, respectively. The compounds in our library with calculated values higher than 1.0 were (*S*)-**18**, (*S*)-**1**, (*S*)-**2**, (*S*)-**6**, (*S*)-**8**, and (*S*)-**13**.

## Conclusions

A variety of *in silico* techniques were evaluated for their potential to speed up the discovery of novel myosin II ATPase inhibitors of the (*S*)-blebbistatin family with a superior target and antitarget profile. First, a variety of methods were assessed for the recovery of active ATPase inhibitors among a series of analogs. Structure-based methods, both without and with crystallographically-observed water molecules, did not perform well. This may be attributed to unaccounted ligand discrimination by steric and temporal restrictions in the path(s) leading toward the binding site. Dissimilarity distances among compounds calculated by extended chemical fingerprints, however, showed good correlations with experimentally determined ATPase activity and offer promise for the selection of actives from hypothetical libraries of analogs. Additional profiling against antitarget proteins with physiological significance for myosin inhibitors (PXR, SULT, androgen receptor, CYPs), and using ADME filters revealed that, for the currently known analogs, compounds (*S*)-**1**, (*S*)-**5**, (*S*)-**12**, (*S*)-**18**, and (*S*)-**19** possess the best overall profile. The techniques and conclusions from this paper may aid in accelerating the discovery of more potent myosin II ATPase inhibitors with appropriate target and antitarget profiles, enabling the development of pharmacological tools for use in motility-related diseases.

## Author contributions

BR provided the (*S*)-blebbistatin analogs, analyzed data and contributed to the research design and writing of the manuscript; RG performed structure-based design to myosin II with GOLD and contributed to the research design and writing of the manuscript; CS contributed to the research design; AG-S performed structure-based design to myosin II with Glide, structure-based design to the five antitargets and androgen receptor, ligand-based design with fingerprints, ADME and PAINS filters, contributed to writing the manuscript, and designed the research project.

### Conflict of interest statement

The authors declare that the research was conducted in the absence of any commercial or financial relationships that could be construed as a potential conflict of interest.

## Footnotes

^1^(2017). Maestro. Schrödinger, LLC: New York. https://www.schrodinger.com/maestro.

^2^(2017). Instant JChem v. 5.6.0. ChemAxon Ltd.: Budapest, Hungary. http://www.chemaxon.com.
