# Supplementary material for: Recovering Actives in Multi-Antitarget and Target Design of Analogs of the Myosin II Inhibitor Blebbistatin
Source: Front Chem. 2018 May 24;6:179. doi: 10.3389/fchem.2018.00179 (PMC5976736; doi:10.3389/fchem.2018.00179)
Supplement: Supplementary file 1 [file Data_Sheet_1.PDF]

Recovering actives in multi-antitarget and target design of analogs of the myosin II inhibitor blebbistatin

Bart I. Roman,[1,2] Rita C. Guedes,[3] Christian V. Stevens,[1,2] and Alfonso T. García-Sosa[4,*]

***Correspondence:**

Dr. Alfonso T. García-Sosa, PhD

alfonsog@ut.ee

Supplementary Information

Contents

Table S1. Sequence alignment for *D. discoideum* (1YV3) and *O. cuniculus* (G1SJQ4)............................2

Table S2. Half-maximum inhibitory concentration (IC50) of (±)-blebbistatin (±)-1 for the ATPase activity of diverse myosins and sequence comparison with selected (S)-blebbistatin contact residues in *Dictyostelium discoideum* myosin II..3

Table S1. Sequence alignment for *D. discoideum* (1YV3) and *O. cuniculus* (G1SJQ4)

```
CLUSTAL O(1.2.4) multiple sequence alignment


tr|G1SJQ4|88-785                  ------------------------------------------------------------     0
1YV3:A|PDBID|CHAIN|SEQUENCE       GNPIHDRTSDYHKYLKVKQGDSDLFKLTVSDKRYIWYNPDPKERDSYECGEIVSETSDSF     60


tr|G1SJQ4|88-785                  -------------------------DKIEDMAMMTHLHEPAVLYNLKERYAAWMIYTYSG     35
1YV3:A|PDBID|CHAIN|SEQUENCE       TFKTVDGQDRQVKKDDANQRNPIKFDGVEDMSELSYLNEPAVFHNLRVRYNQDLIYTYSG     120
                                                           * :***: :::*:****::**: **   :******

tr|G1SJQ4|88-785                  LFCVTVNPYKWLPVYNPEVVTAYRGKKRQEAPPHIFSISDNAYQFMLTDRENQSILITGE     95
1YV3:A|PDBID|CHAIN|SEQUENCE       LFLVAVNPFKRIPIYTQEMVDIFKGRRRNEVAPHIFAISDVAYRSMLDDRQNQSLLITGE     180
                                  ** *:***:* :*:*. *:*  ::*::*:*. ****:*** **: ** **:***:*****

tr|G1SJQ4|88-785                  SGAGKTVNTKRVIQYFATIAVTGDKKKEEATSGKMQGTLEDQIISANPLLEAFGNAKTVR     155
1YV3:A|PDBID|CHAIN|SEQUENCE       SGAGKTENTKKVIQYLASVAGRNQ--------ANGSGVLEQQILQANPILEAFGNAKTTR     232
                                  ****** ***:****:*::*  .:        .: .*.**:**:.***:*********.*

tr|G1SJQ4|88-785                  NDNSSRFGKFIRIHFGTTGKLASADIETYLLEKSRVTFQLKAERSYHIFYQITSNKKPEL     215
1YV3:A|PDBID|CHAIN|SEQUENCE       NNNSSRFGKFIEIQFNSAGFISGASIQSYLLEKSRVVFQSETERNYHIFYQLLAGATAEE     292
                                  *:*********.*:*.::* ::.*.*::********.** ::**.******: :. . *

tr|G1SJQ4|88-785                  IEMLLITTNPYDYPFV-SQGEISVASIDDQEELMATDSAIDILGFTNEEKVSIYKLTGAV     274
1YV3:A|PDBID|CHAIN|SEQUENCE       KKALHL-AGPESFNYLNQSGCVDIKGVSDSEEFKITRQAMDIVGFSQEEQMSIFKIIAGI     351
                                   : * : :.* .: :: ..* :.: .:.*.**:  * .*:**:**::**::**:*: ..:

tr|G1SJQ4|88-785                  MHYGNMKFKQKQREEQAEPDGTEVADKAAYLQGLNSADLLKALCYPRVKVGNEFVTKGQT     334
1YV3:A|PDBID|CHAIN|SEQUENCE       LHLGNIKFEKGAGEGAVLKDKTA-LNAASTVFGVNPSVLEKALMEPRILAGRDLVAQHLN     410
                                  :* **:**::   *  .  * *   : *: : *:* : * ***  **: .*.::*::  .

tr|G1SJQ4|88-785                  VEQVTNAVGALAKAVYEKMFLWMVTRINQQLDTKQPRQYFIGVLDIAGFEIFDFNSLEQL     394
1YV3:A|PDBID|CHAIN|SEQUENCE       VEKSSSSRDALVKALYGRLFLWLVKKINNVLC-QERKAYFIGVLDISGFEIFKVNSFEQL     469
                                  **: :.: .**.**:* ::***:*.:**: *  :: : ********:*****..**:***

tr|G1SJQ4|88-785                  CINFTNEKLQQFFNHHMFVLEQEEYKKEGIEWTFIDFGMDLAACIELIE--KPMGIFSIL     452
1YV3:A|PDBID|CHAIN|SEQUENCE       CINYTNEKLQQFFNHHMFKLEQEEYLKEKINWTFIDFGLDSQATIDLIDGRQPPGILALL     529
                                  ***:************** ****** ** *:*******:*  * *:**:  :* **:::*

tr|G1SJQ4|88-785                  EEECMFPKATDTSFKNKLYDQHLGKSANFQKPKVVKGKVEAHFSLIHYAGVVDYNITGWL     512
1YV3:A|PDBID|CHAIN|SEQUENCE       DEQSVFPNATDNTLITKLHSHFSKKNAKYEEPRFSK----TEFGVTHYAGQVMYEIQDWL     585
                                  :*:.:**:***.:: .**:.:.  *.*::::*:. *    :.*.: **** * *:* .**

tr|G1SJQ4|88-785                  EKNKDPLNETVVGLYQKSSLKTLAFLFSGAQTAEAEASGGAKKGGKKKGSSFQTVSALFR     572
1YV3:A|PDBID|CHAIN|SEQUENCE       EKNKDPLQQDLELCFKDSSDNVVTKLFNDPNI----------ASRAKKGANFITVAAQYK     635
                                  *******:: :   ::.** :.:: **.. :            .  ***:.* **:* ::

tr|G1SJQ4|88-785                  ENLNKLMTNLRSTHPHFVRCIIPNETKTPGAMEHELVLHQLRCNGVLEGIRICRKGFPSR     632
1YV3:A|PDBID|CHAIN|SEQUENCE       EQLASLMATLETTNPHFVRCIIPNNKQLPAKLEDKVVLDQLRCNGVLEGIRITRKGFPNR     695
                                  *:* .**:.*.:*:**********:.: *. :*.::**.************* *****.*

tr|G1SJQ4|88-785                  ILYADFKQRYKVLNASAIPEGQYIDSKKASEKLLGSIDIDHTQYKFGHTKVFFKAGLLGL     692
1YV3:A|PDBID|CHAIN|SEQUENCE       IIYADFVKRYYLLAPNVPR--DAEDSQKATDAVLKHLNIDPEQYRFGITKIFFRAGQLAR     753
                                  *:**** :** :*  ..    :  **:**:: :*  ::**  **:** **:**:** *.

tr|G1SJQ4|88-785                  LEEMRD---      698
1YV3:A|PDBID|CHAIN|SEQUENCE       IEEARELPN      762
                                  :** *:
```

Table S2. Half-maximum inhibitory concentration (IC50) of (±)-blebbistatin (±)-1 for the ATPase activity of diverse myosins and sequence comparison with selected (S)-blebbistatin contact residues in *Dictyostelium discoideum* myosin II.

Table 1. IC_{50} of (±)-blebbistatin (±)-1 for the ATPase activity of diverse myosins and sequence comparison with selected (*S*)-blebbistatin contact residues in *Dictyostelium discoideum* myosin II.[1]

Species	Myosin type	IC_{50} [a] (μM)	Selected (*S*)-blebbistatin contact residues[b]				Reference(s)
D. discoideum	II	4.9–13[a]	Gly240	Leu262	Ser456	Ile455	3,4,5,8,9,13
H. sapiens	Non-muscle IIA	4–14[a]	Gly	Leu	Ala	Ile	2,3,14
O. cuniculus	Skeletal-muscle II	0.22–4.32[a]	Gly	Leu	Ala	Ile	3,5,6,8,9,10,11,12

[a] Data obtained with (*S*)-blebbistatin (*S*)-**1** were recalculated to mixtures containing 50% (*S*)-blebbistatin (*S*)-**1** and 50% (*R*)-blebbistatin (*R*)-**1**, assuming that (*R*)-blebbistatin (*R*)-**1** would not contribute to inhibition. [b] Sequence alignments were performed on the UniProt website (accession numbers are P08799, P35579, Q28641, respectively).[15]

1. Roman, B. I.; Verhasselt, S.; Stevens, C. V. The Medicinal Chemistry and Use of Myosin II Inhibitor (S)-Blebbistatin and Its Derivatives. *J. Med. Chem.* **2018**, submitted.
2. Straight, A. F.; Cheung, A.; Limouze, J.; Chen, I.; Westwood, N. J.; Sellers, J. R.; Mitchison, T. J. Dissecting temporal and spatial control of cytokinesis with a myosin II Inhibitor. *Science* **2003**, *299*, 1743-1747.
3. Limouze, J.; Straight, A. F.; Mitchison, T.; Sellers, J. R. Specificity of blebbistatin, an inhibitor of myosin II. *J. Muscle Res. Cell Motil.* **2004**, *25*, 337-341.
4. Lucas-Lopez, C.; Patterson, S.; Blum, T.; Straight, A. F.; Toth, J.; Slawin, A. M. Z.; Mitchison, T. J.; Sellers, J. R.; Westwood, N. J. Absolute stereochemical assignment and fluorescence tuning of the small molecule tool, (–)-blebbistatin. *Eur. J. Org. Chem.* **2005**, 1736-1740.
5. Tóth, J. *Functional characterization of a novel myosin and a novel myosin inhibitor*; Ph.D. Dissertation, Eötvös Loránd University, Budapest, Hungary, **2006**.
6. Képiró, M.; Várkuti, B. H.; Végner, L.; Vörös, G.; Hegyi, G.; Varga, M.; Málnási-Csizmadia, A. para-Nitroblebbistatin, the non-cytotoxic and photostable myosin II inhibitor. *Angew. Chem., Int. Ed.* **2014**, *53*, 8211-8215.

7. Képiró. M. Azidation Technology: From Photoaffinity Labeling to Molecular Tattooing; Ph.D. Dissertation, Eötvös Loránd University, Budapest, Hungary, **2014**.

8. Várkuti, B. H.; Képiró, M.; Horváth, I. Á.; Végner, L.; Ráti, S.; Zsigmond, Á.; Hegyi, G.; Lenkei, Z.; Varga, M.; Málnási-Csizmadia, A. A highly soluble, non-phototoxic, non-fluorescent blebbistatin derivative. *Sci. Rep.* **2016**, *6*: 26141.

9. Képiró, M.; Várkuti, B. H.; Bodor, A.; Hegyi, G.; Drahos, L.; Kovács, M.; Málnási-Csizmadia, A. Azidoblebbistatin, a photoreactive myosin inhibitor. *Proc. Natl. Acad. Sci. USA* **2012**, *109*, 9402-9407.

10. Verhasselt, S.; Roman, B. I.; De Wever, O.; Van Hecke, K.; Van Deun, R.; Bracke, M. E.; Stevens, C. V. Discovery of (S)-3'-hydroxyblebbistatin and (S)-3'-aminoblebbistatin: polar myosin II inhibitors with superior research tool properties. *Org. Biomol. Chem.* **2017**, *15*, 2104-2118.

11. Verhasselt, S.; Roman, B. I.; Bracke, M. E.; Stevens, C. V. Improved synthesis and comparative analysis of the tool properties of new and existing D-ring modified (S)-blebbistatin analogs. *Eur. J. Med. Chem.* **2017**, *136*, 85-103

12. Verhasselt, S. *Development of Novel Blebbistatin Derivatives in the Quest for Improved Non-Muscle Myosin II Inhibitors*; Ph.D. Dissertation, Ghent University, Ghent, Belgium, **2017**.

13. Shu, S.; Liu, X.; Korn, E. D. Blebbistatin and blebbistatin-inactivated myosin II inhibit myosin II-independent processes in Dictyostelium. *Proc. Natl Acad. Sci. USA* **2005**, 102, 1472-1477.

14. Zhang, H.-M.; Ji, H.-H.; Ni, T.; Ma, R.-N.; Wang, A.; Li, X.-d. Characterization of Blebbistatin Inhibition of Smooth Muscle Myosin and Nonmuscle Myosin-2. *Biochemistry* **2017**, 56, 4235-4243.

The UniProt Knowledgebase. www.uniprot.org/uniprot/ (accessed March
